# 3Dscript.server: true server-side 3D animation of microscopy images using a natural language-based syntax

**DOI:** 10.1093/bioinformatics/btab462

**Published:** 2021-06-21

**Authors:** Benjamin Schmid, Philipp Tripal, Zoltán Winter, Ralph Palmisano

**Affiliations:** Optical Imaging Centre Erlangen, University of Erlangen-Nuremberg, 91058 Erlangen, Germany; Optical Imaging Centre Erlangen, University of Erlangen-Nuremberg, 91058 Erlangen, Germany; Optical Imaging Centre Erlangen, University of Erlangen-Nuremberg, 91058 Erlangen, Germany; Optical Imaging Centre Erlangen, University of Erlangen-Nuremberg, 91058 Erlangen, Germany; Optical Imaging Centre Erlangen, University of Erlangen-Nuremberg, 91058 Erlangen, Germany

## Abstract

**Summary:**

Creating 3D animations from microscopy data is computationally expensive and requires high-end hardware. We therefore developed 3Dscript.server, a 3D animation software that runs as a service on dedicated, shared workstations. Using 3Dscript as the underlying rendering engine, it offers unique features not found in existing software: rendering is performed completely server-side. The target animation is specified on the client without the rendering engine, eliminating any hardware requirements client-side. Still, defining an animation is intuitive due to 3Dscript’s natural language-based animation description. We implemented a new OMERO web app to utilize 3Dscript.server directly from the OMERO web interface; a Fiji client to use 3Dscript.server from Fiji for integration into image processing pipelines; and batch scripts to run 3Dscript.server on compute clusters for large-scale visualization projects.

**Availability and implementation:**

Source code and documentation is available at https://github.com/bene51/omero_3Dscript, https://github.com/bene51/3Dscript.server and https://github.com/bene51/3Dscript.cluster.

**Supplementary information:**

Supplementary data are available at *Bioinformatics* online.

## 1. Introduction

Today, there is an increasing endeavor to enable remote work wherever possible. Still, for creating 3D animations, users need physical access to a dedicated rendering computer, as the only alternative, screen-sharing, often lacks the required responsiveness. To our knowledge, no software exists that runs as a service, to which users can submit animation jobs from remote. The reason for this gap is the way existing software creates 3D animations: The user adjusts the state of the 3D scene, including orientation, color, etc., for different time points, in keyframes. The rendering engine then creates a movie by interpolating between them. Keyframes therefore constitute a necessary input for the animation service. Creating them, however, is interactive and therefore requires the rendering engine and also the corresponding hardware on the client.

Recently, we have presented 3Dscript (Schmid *et al.*, 2019), where textual instructions, written in a natural language-based syntax (‘From frame 0 to frame 100 rotate by 70 degrees horizontally’), describe and define arbitrarily complex animations and thereby replace keyframes. 3Dscript fulfills two criteria that make it ideally suited for a rendering framework based on a client-server architecture: animations are defined as human-readable text that gives a good idea of the rendering result even without rendering engine and preview, and this text is composed without any hardware requirements (as it is text only). We used this fact to develop a framework that for the first time creates 3D animations purely server-side.

Here, we introduce 3Dscript.server, which runs a 3D rendering server, implemented as an ImageJ/Fiji plugin (Rueden *et al.*, 2017; Schindelin *et al.*, 2012; Schneider *et al.*, 2012) with 3Dscript as the underlying framework. We present three client implementations, (i) 3Dscript.client, a Fiji client for seamless integration into image analysis pipelines. (ii) OMERO.3Dscript, a web application for the image management software OMERO (Allan *et al.*, 2012) and (iii) 3Dscript.cluster, to run large-scale 3D animation projects on a high-performance compute cluster (Supplementary Fig. 1).

Closest related to the current work is ClearVolume (Royer *et al.*, 2015), for remotely observing in real-time data acquisition on a volumetric microscope; FPBioimage (Fantham and Kaminski, 2017), for collaborative 3D visualization of biomedical images in a web browser; and BigDataViewer (Pietzsch *et al.*, 2015), for visualizing arbitrarily sized datasets. In all three applications, input datasets are loaded from a server but rendered client-side.

## 2. Results

### 3Dscript.server: the server module

To set up a shared server environment, 3Dscript.server is first installed on one or more capable workstations in the local network (Supplementary Video S1). The server runs as a Fiji plugin and offers a single-click configuration to start automatically when the operating system boots. 3Dscript.server assumes its input data to be stored centrally, so that it is not loaded from slow, possibly wireless, client connections. Instead, data are retrieved either from an image management system such as OMERO or from a shared file system, operated within a fast institutional network. Because a rendering job will typically demand all graphics processing unit (GPU) resources, received jobs are collected in a queue and processed sequentially. If the input data is loaded from OMERO, the result is uploaded as a compressed video file as an attachment to the input image. In case of a shared file system, it is uploaded to the directory containing the input data (Supplementary Fig. 2).

### 3Dscript.client: accessing 3Dscript.server from a Fiji client

3Dscript.client runs as a Fiji plugin and can distribute rendering to several user-specified servers simultaneously. It conveniently detects all available servers in the local network (Supplementary Videos S2 and S3). We implemented 3Dscript.client such that it can be integrated in macros and scripts as part of comprehensive workflows.

### OMERO.3Dscript: accessing 3Dscript.server through OMERO.web

**Fig. 1.**
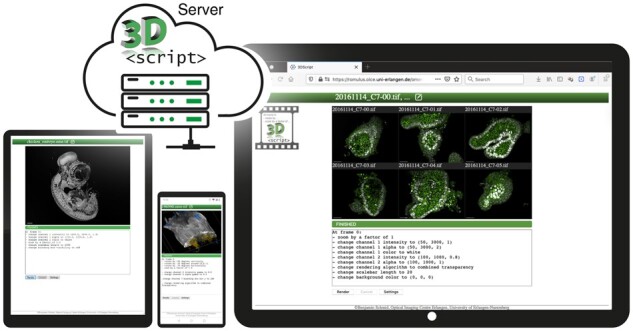
Platform- and hardware-independent animations through server-side 3D rendering with 3Dscript.server. OMERO.3Dscript is implemented as an OMERO web application, accessing 3Dscript.server for the rendering. Its front end runs in a web browser on desktop and mobile devices, without hardware requirements, as rendering is performed server-side. Entire collections of images can be rendered together with identical parameters. Example data: chicken embryo nervous system imaged with a mesoSPIM lightsheet microscope (Voigt *et al.*, 2019), mouse paw acquired on an ultramicroscope lightsheet system, mouse small intestinal organoids recorded on a spinning disk microscope (Bardenbacher *et al.*, 2019)

We developed OMERO.3Dscript (Fig. 1, Supplementary Video S4, Supplementary Material) as a web application for OMERO, the most popular image management solution in the field of scientific imaging to date. For users, OMERO.3Dscript minimizes the effort to create high-quality and reproducible 3D animations: It runs directly in a web browser, and users create 3D animations from within their familiar OMERO web environment. An auto-completion enabled text field supports users in composing the animation text. Once rendering is started, OMERO.3Dscript continuously requests the rendering progress from the server and updates the website accordingly. The rendered video is uploaded to OMERO as an attachment to the input dataset in addition to being displayed in the web interface. OMERO.3Dscript processes entire collections of images at once (Supplementary Video S5). It can run on the same machine that stores the imaging data, which avoids large input data transfer completely. There are no hardware requirements for the client, and since OMERO.3Dscript’s frontend runs in a browser, clients can even connect from mobile devices (Supplementary Video S6). With the animation text being written in natural English language, it may even be entered conveniently using speech recognition.

### 3Dscript.cluster: running 3Dscript.server on an HPC cluster

To create 3D animations on a larger scale, 3Dscript.server also runs on compute clusters. Different time points of time-lapse data or different datasets of image collections are processed in parallel on distinct nodes, which reduces the processing time significantly. We developed scripts for submitting rendering tasks to the ‘Emmy’ compute cluster of the University of Erlangen-Nuremberg, which includes 10 nodes with at least one GPU required for 3Dscript. The provided batch scripts are written for the Torque job management system (Adaptive Computing Inc.) but are easily adapted to different systems.

With 3Dscript.server, it is finally possible to create high-quality scientific 3D animations ‘on-the-go’, e.g. on a tablet PC while discussing a poster on a conference, instead of sitting in front of a high-end workstation.

## Supplementary Material

btab462_Supplementary_Data

## References

[btab462-B1] Allan C. et al (2012) OMERO: flexible, model-driven data management for experimental biology. Nat. Methods, 9, 245–253. 22373911 10.1038/nmeth.1896 PMC3437820

[btab462-B2] Bardenbacher M. et al (2019) Permeability analyses and three dimensional imaging of interferon gamma-induced barrier disintegration in intestinal organoids. Stem Cell Res., 35, 101383. 30776676 10.1016/j.scr.2019.101383

[btab462-B3] Fantham M. , Kaminski C.F. (2017) A new online tool for visualization of volumetric data. Nat. Photonics, 11, 69.

[btab462-B4] Pietzsch T. et al (2015) BigDataViewer: visualization and processing for large image data sets. Nat. Methods, 12, 481–483. 26020499 10.1038/nmeth.3392

[btab462-B5] Royer L.A. et al (2015) ClearVolume: open-source live 3D visualization for light-sheet microscopy. Nat. Methods, 12, 480–481. 26020498 10.1038/nmeth.3372

[btab462-B6] Rueden C.T. et al (2017) ImageJ2: imageJ for the next generation of scientific image data. BMC Bioinformatics, 18, 529. 29187165 10.1186/s12859-017-1934-z PMC5708080

[btab462-B7] Schindelin J. et al (2012) Fiji: an open-source platform for biological-image analysis. Nat. Methods, 9, 676–682. 22743772 10.1038/nmeth.2019 PMC3855844

[btab462-B8] Schmid B. et al (2019) 3Dscript: animating 3D/4D microscopy data using a natural-language-based syntax. Nat. Methods, 16, 278–280. 30886414 10.1038/s41592-019-0359-1

[btab462-B9] Schneider C.A. et al (2012) NIH Image to ImageJ: 25 years of image analysis. Nat. Methods, 9, 671–675. 22930834 10.1038/nmeth.2089 PMC5554542

[btab462-B10] Voigt F.F. et al (2019) The mesoSPIM initiative: open-source light-sheet microscopes for imaging cleared tissue. Nat. Methods, 16, 1105–1108. 31527839 10.1038/s41592-019-0554-0 PMC6824906

