# Supplementary material for: 3Dscript.server: true server-side 3D animation of microscopy images using a natural language-based syntax
Source: Bioinformatics. 2021 Jun 21;37(24):4901–2. doi: 10.1093/bioinformatics/btab462 (PMC8665743; doi:10.1093/bioinformatics/btab462)
Supplement: btab462_Supplementary_Data [file btab462_supplementary_data.zip › Supplement_Schmid2021.pdf]

Supplementary Material

3Dscript.server: True server-side 3D animation of microscopy images using a natural language-based syntax

Benjamin Schmid [1,*], Philipp Tripal [1], Zoltán Winter [1] and Ralph Palmisano [1]

[1]Optical Imaging Centre Erlangen, University of Erlangen-Nuremberg, Erlangen, Germany.
[*]To whom correspondence should be addressed.

1 OMERO.3Dscript demo guide

- Open the OMERO web interface in a browser. We set up a demo server which is accessible under https://euclid.oice.uni-erlangen.de.
  Please note that OMERO.3Dscript works on in all common browsers (e.g. Mozilla Firefox, Microsoft Edge, Safari, Google Chrome, ...), but currently doesn't support Microsoft Internet Explorer.
- In the left panel, click on *3Dscript>Organoids*
- Right-click *20161114_C7-00.tif* and choose *Open With...>3Dscript*
- Click on *Render* to render the preset animation
- Optionally, change the animation description text. More information can be found
  - in the 3Dscript publication, https://www.nature.com/articles/s41592-019-0359-1
  - in the 3Dscript user documentation on the GitHub wiki, in particular at https://github.com/bene51/3Dscript/wiki/The-animation-language and https://github.com/bene51/3Dscript/wiki/Example---step-by-step.
  - on the 3Dscript gallery page, https://bene51.github.io/3Dscript/gallery.html.

To render the same animation for multiple data sets:

- Go ck to the OMERO web client, select more of the organoids, e.g. by `Ctrl/Cmd` clicking, click the chain symbol in the top right corner of the web UI and copy the entire link.
- Switch to the 3Dscript tab
- Click the *Edit* symbol next to the image name
- Paste the link for *New image ID* and click OK.
- Click on *Render*.
- Once done, click on *Play all* to play the rendered animations side by side.

2 Implementation details

3Dscript.server

3Dscript.server is implemented in Java as a Fiji plugin. A producer thread listens on a network socket for incoming commands to start or cancel rendering or retrieve information about the current rendering task. Upon receiving a start render command, a new rendering job is initialized and appended to a FIFO queue. A consumer thread polls jobs from the queue and processes them sequentially using 3Dscript. Input data is either loaded from a shared file system using the jcifs java library (https://github.com/codelibs/jcifs) or from an OMERO server using the OMERO java bindings. To automatically discover machines running 3Dscript.server in the local network, 3Dscript.server creates a multicast socket and joins a multicast group to receive UDP datagram packets (a discovery request) from discovering clients, upon which it returns its IP address.

3Dscript.client

3Dscript.client is implemented in Java as a Fiji plugin. It sends commands to start or cancel rendering to a 3Dscript.server via a network socket. After rendering is started, 3Dscript.client continuously polls the current rendering status from the server, until rendering is finished, canceled or an error occurred. To automatically identify machines running 3Dscript.server in the local network, 3Dscript.client sends a discovery request datagram packet to the multicast group to receive the IP addresses from connected servers.

OMERO.3Dscript

OMERO.3Dscript is implemented in Python as an OMERO.web app using the Django Python web framework (https://www.djangoproject.com/). It sends rendering and status retrieval commands over a network port to 3Dscript.server, which runs either on the same machine as OMERO.web or remotely. The web frontend is implemented in HTML and JavaScript, using the backbone.js framework (https://backbonejs.org/), and optimized for mobile devices. Once a rendering job is started, the rendering state is continuously polled using AJAX requests and displayed to the user. The OMERO.3Dscript web frontend includes a text area for the rendering script that features autocompletion, implemented client-side in JavaScript using jQuery UI (https://jqueryui.com). Once rendering is complete, 3Dscript.server uploads the resulting video and a poster image as an attachment of the input image to the OMERO server, from where it can be downloaded at any time. Additionally, the poster image is displayed on the web frontend. Once clicked, the entire video is loaded. Lazy-loading helps to reduce data transfer, which plays a role when operating OMERO.3Dscript from a mobile device over a metered internet connection. OMERO.3Dscript was tested using the web automation tool playwright (https://github.com/microsoft/playwright).

3 Supplementary Figure 1

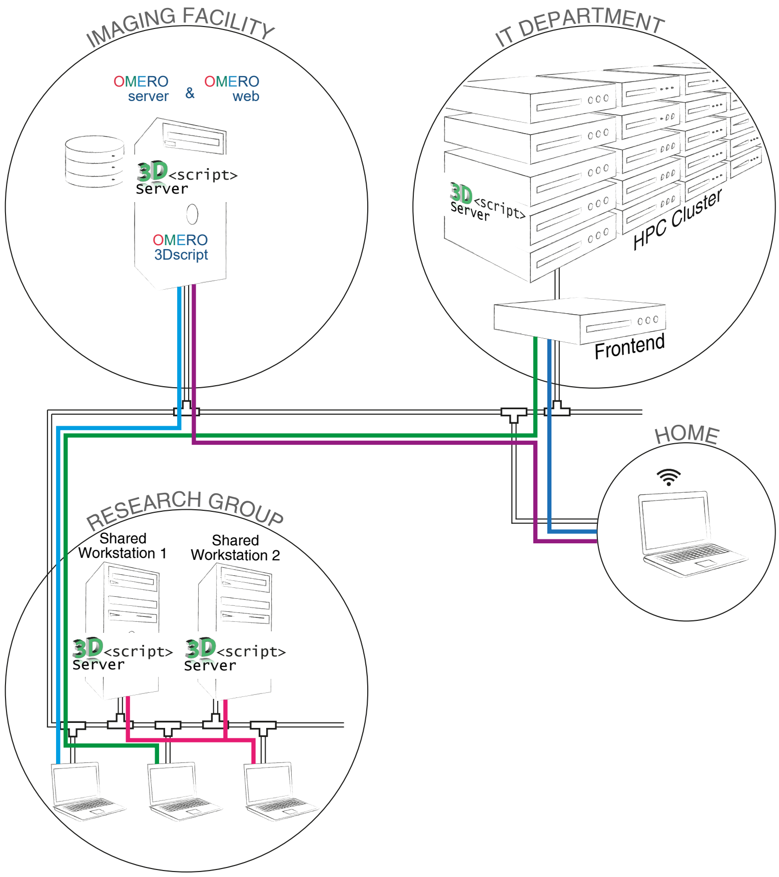

Supplementary Figure 1: Platform- and hardware-independent animations through server-side 3D rendering with 3Dscript.server. 3Dscript.server can run standalone, on an OMERO server or on a compute cluster. The Fiji client delegates processing to standalone servers, but also to 3Dscript.servers on an OMERO server. OMERO.3Dscript is implemented as an OMERO web application. For processing, it can either use a 3Dscript.server running on the same machine or a standalone 3Dscript.server. 3Dscript.server running on a cluster is executed via the cluster's job management system by an appropriate job script.

4 Supplementary Figure 2

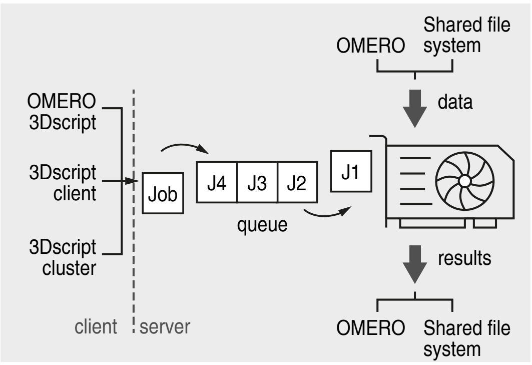

Supplementary Figure 2: 3Dscript.server collects rendering jobs from clients in a queue and renders them sequentially. Input data is retrieved from either an OMERO.server or from a shared file system, and the resulting videos are uploaded accordingly.

5 Supplementary Video 1

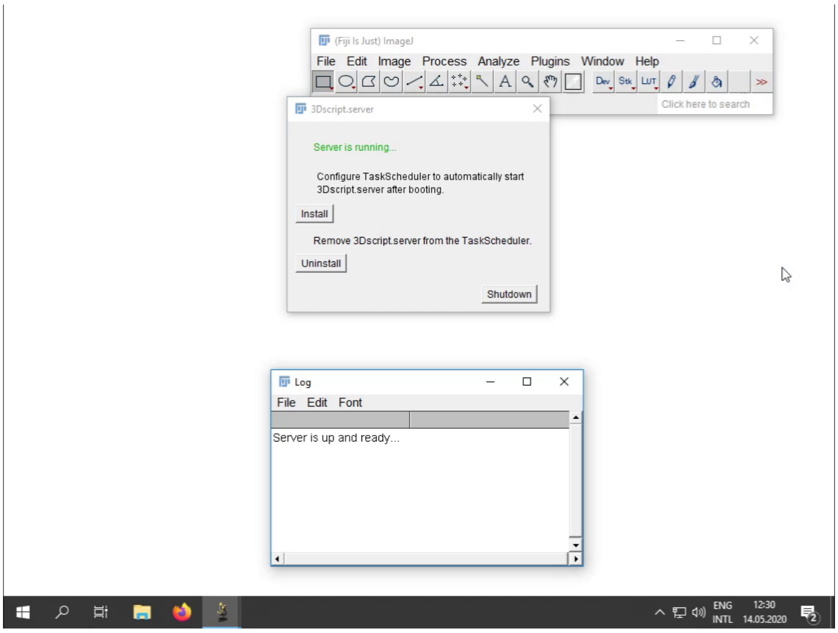

Supplementary Video 1: Running the 3Dscript.server Fiji plugin. It is trivially started like any other Fiji plugin. On Microsoft Windows, the Task Scheduler can automatically be configured from within the plugin to start Fiji and the 3Dscript.server plugin after the computer booted. https://youtu.be/ZNu-FVaR2AA

6 Supplementary Video 2

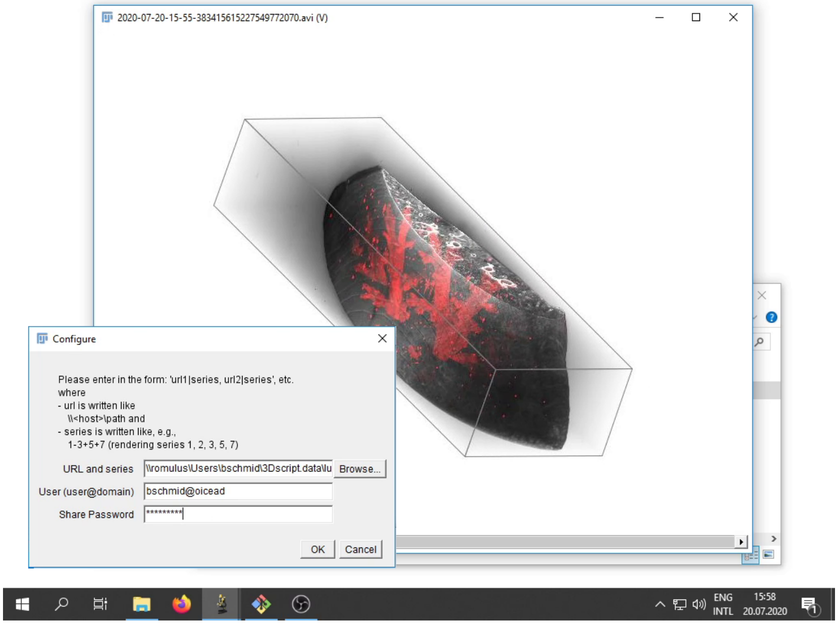

Supplementary Video 2: — Running the 3Dscript.client Fiji plugin with data from a shared file system. The plugin connects and submits rendering jobs to one or more running 3Dscript.servers, which are chosen by the user. 3Dscript.servers in the local network can be discovered automatically. The dataset shows a mouse lung imaged on an ultramicroscope lightsheet system. https://youtu.be/qcVR0et8cMM

7 Supplementary Video 3

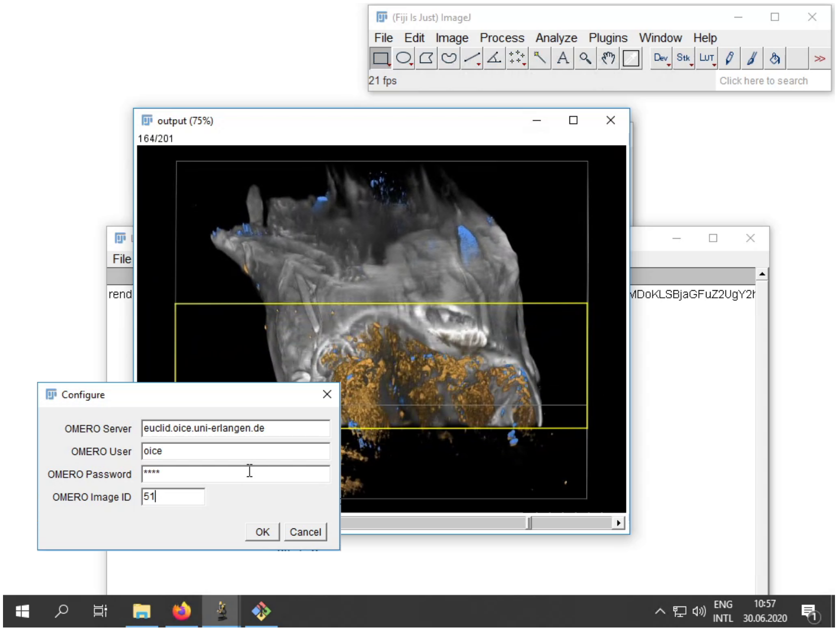

Supplementary Video 3: Running the 3Dscript.client Fiji plugin with data from an OMERO server. The plugin connects and submits rendering jobs to one or more running 3Dscript.servers, which are chosen by the user. 3Dscript.servers in the local network can be discovered automatically. The dataset shows a mouse paw imaged on an ultramicroscope lightsheet system. https://youtu.be/k6vAqK8qgJo

8 Supplementary Video 4

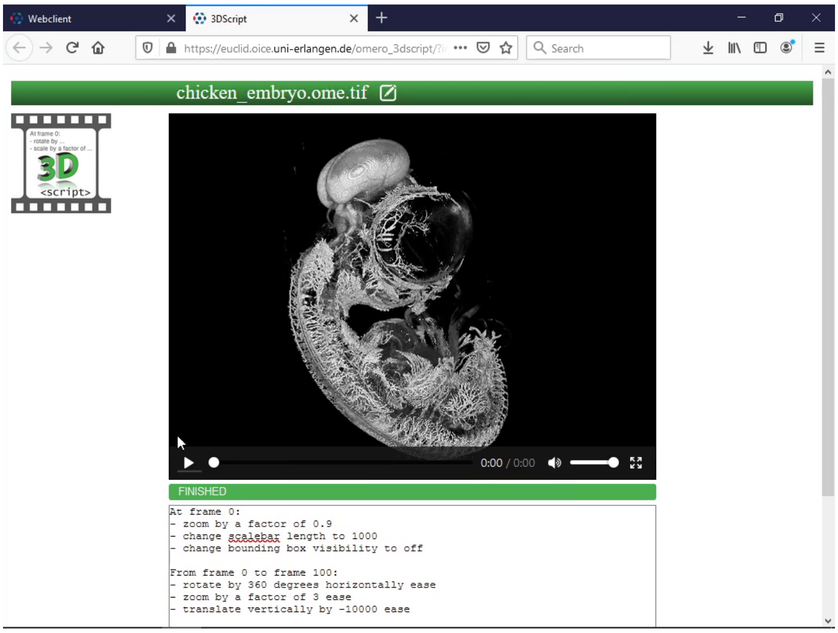

Supplementary Video 4: Interactively rendering a single dataset from the OMERO.3Dscript web frontend. The website is opened from the main OMERO.web page. Animation text is entered in a dedicated text area, supported by auto-completion. Once rendering is started, the current state and progress is polled to update the website continuously until finished. The resulting animation video is saved as an attachment to the input image in OMERO, from where it can be downloaded at any time. The dataset shows a chicken embryo's nervous system imaged with a mesoSPIM lightsheet microscope (Voigt *et al.*, 2019). https://youtu.be/a-OvZ_F6uU8

9 Supplementary Video 5

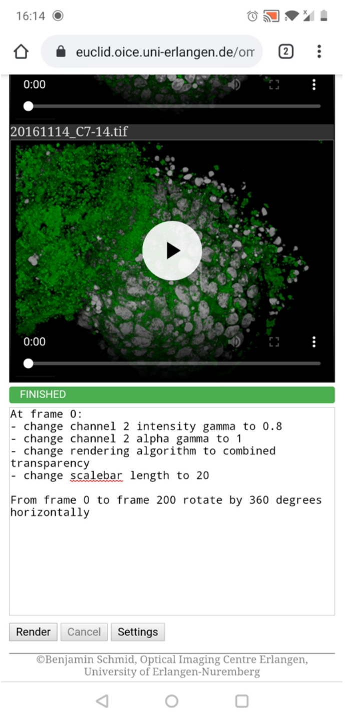

Supplementary Video 5: Rendering multiple datasets from the OMERO.3Dscript web frontend using a smartphone. The OMERO.3Dscript website is optimized for mobile devices. Typing the animation text on touch keyboards particularly benefits from auto-completion. The example data show mouse small intestinal organoids recorded on a spinning disk microscope (Bardenbacher *et al.*, 2019). https://youtu.be/-xMsam29BPo

10 Supplementary Video 6

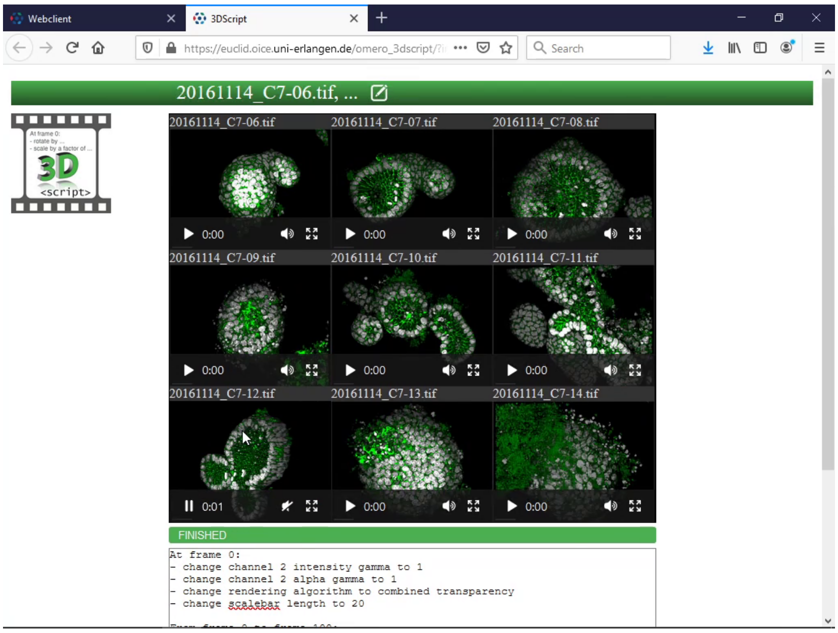

Supplementary Video 6: Once the animation text is optimized on a single dataset, it can conveniently be used to render an arbitrary collection of images together. The example data show mouse small intestinal organoids recorded on a spinning disk microscope (Bardenbacher *et al.*, 2019). https://youtu.be/PzShSyIIEnc

References

Bardenbacher, M., Ruder, B., Britzen-Laurent, N., Schmid, B., Waldner, M., Naschberger, E., Scharl, M., Müller, W., Günther, C., Becker, C., Stürzl, M., and Tripal, P. (2019). Permeability analyses and three dimensional imaging of interferon gamma-induced barrier disintegration in intestinal organoids. *Stem Cell Research*, **35**.

Voigt, F. F., Kirschenbaum, D., Platonova, E., Pagès, S., Campbell, R. A., Kastli, R., Schaettin, M., Egolf, L., van der Bourg, A., Bethge, P., Haenraets, K., Frézel, N., Topilko, T., Perin, P., Hillier, D., Hildebrand, S., Schueth, A., Roebroeck, A., Roska, B., Stoeckli, E. T., Pizzala, R., Renier, N., Zeilhofer, H. U., Karayannis, T., Ziegler, U., Batti, L., Holtmaat, A., Lüscher, C., Aguzzi, A., and Helmchen, F. (2019). The mesoSPIM initiative: open-source light-sheet microscopes for imaging cleared tissue. *Nature Methods*, **16**(11), 1105–1108.
